# Adipokines in Psoriatic Arthritis Patients: The Correlations with Osteoclast Precursors and Bone Erosions

**DOI:** 10.1371/journal.pone.0046740

**Published:** 2012-10-29

**Authors:** Yu Xue, Li Jiang, Qingqing Cheng, Haiyan Chen, Yiyun Yu, Yinda Lin, Xue Yang, Ning Kong, Xiaoxia Zhu, Xue Xu, Weiguo Wan, Hejian Zou

**Affiliations:** 1 Department of Rheumatology, Huashan Hospital, Institute of Rheumatology, Immunology and Allergy, Fudan University, Shanghai, China; 2 Department of Rheumatology, Linyi People's Hospital, Shandong, China; Oklahoma State University, United States of America

## Abstract

Significant bone remodeling with disordered osteoclastogenesis has been implicated in the pathogenesis of psoriatic arthritis (PsA). And there is a high prevalence of the metabolic syndrome (MS) in PsA patients. Adipokines, especially leptin and adiponectin, have recently been reported to be involved in the development and regulation of some autoimmune diseases. In this study, we examined the alternation of circulating osteoclastogenesis related cytokines [tumor necrosis factor-α (TNF-α), osteoprotegerin (OPG) and receptor activator of nuclear factor-κB ligand (RANKL)] and adipokines (leptin, adiponectin, resistin, chemerin, omentin) in PsA patients, and analysed the correlations between these factors and osteoclast precursors numbers, radiographic damage scores, and disease activity index. 41 PsA patients, 20 psoriasis patients, and 24 healthy controls were recruited. Blood samples were obtained for detecting the levels of TNF-α, OPG, RANKL and the adipokines. The numbers of osteoclast precursors (OCs) in peripheral blood were assessed. Radiographs of affected joints in PsA patients were scored for erosion, joint-space narrowing, osteolysis, and new bone formation. Compared with healthy controls, patients with PsA had higher TNF-α, RANKL, OCs, leptin and omentin but lower adiponectin and chemerin. Increased serum levels of TNF-α, RANKL, leptin, and omentin were positively correlated with OCs numbers. In contrast, serum adiponectin levels were decreased in PsA patients and negatively correlated with OCs numbers. TNF-α, RANKL and leptin were positively correlated with Psoriatic Arthritis Joint Activity Index (PsAJAI). Only TNF-α was positively correlated with radiographic damage scores. Our data demonstrated that systemic expression of soluble mediators of osteoclastogenesis and adipokines were disordered in PsA. Certain adipokines were elevated in the circulation of patients with PsA and might contribute to pathogenesis of arthritis. Prospective studies will be of interest to determine the pluripotent effects of adipokines on osteoclastogenesis in chronic inflammatory rheumatic diseases. Future studies may lead to novel therapeutic strategies.

## Introduction

Psoriatic arthritis (PsA) is an inflammatory arthritis, which is typically associated with psoriasis and psoriatic nail disease. It has both peripheral articular manifestations (including synovitis, dactylitis, and enthesitis) and axial skeletal involvement. A range of bone pathologies were observed in patients with PsA including aberrant bone loss and new bone formation [1], [2]. Now, it is apparent that PsA is more aggressive than previously thought and the majority of patients with PsA experience a chronic, progressive course. Approximately one-fifth of patients with PsA develop to a destructive, disabling form of arthritis over time. Two main cell types are involved in bone remodeling: osteoclasts and osteoblasts. RANKL-mediated osteoclastogenesis has been implicated in the pathogenesis of bone resorption in PsA [3]–[6].

Patients with chronic inflammatory diseases are prone to develop metabolic syndrome (MS). A recent study demonstrated that patients with PsA, but not Rheumatoid Arthritis (RA) or Ankylosing Spondylitis (AS), had significantly higher prevalence of the metabolic syndrome compared to the general population [7]. Adipokines, cytokines derived from adipose tissues, are key players in the pathogenesis of metabolic syndrome. They not only contribute to the regulation of body functions such as insulin-mediated processes, lipid and glucose metabolism, vascular changes and coagulation, but also participate in chronic inflammation. Leptin and adiponectin have recently been found involved in the development and regulation of autoimmune diseases [8], [9].

Because of the high prevalence of MS in PsA patients, we are interested in the impacts of adipokines on the psoriatic arthritis etiology, osteoclastogenesis and bone remodeling. In this study, we investigated alternation of circulating osteoclastogenesis related cytokines (TNF-α, OPG and RANKL) and adipokines (leptin, adiponectin, resistin, chemerin, omentin) in psoriatic arthritis patients, and their correlation with osteoclast precursors, radiographic damage scores and disease activity index.

## Materials and Methods

### Patients and clinical assessments

This study was approved by the Ethics Committee of Huashan Hospital, Fudan University. All the patients provided written informed consent. Forty-one patients with PsA were recruited from rheumatology clinics in Huashan Hospital. All patients with PsA met the Classification of Psoriatic Arthritis (CASPAR) criteria for PsA [10]. In addition, two control groups were studied: patients with psoriasis but no arthritis (n = 20) and healthy volunteers with no psoriasis or arthritis (n = 24). Psoriasis control and healthy control participants had no previous diagnosis of arthritis and no evidence of synovitis, enthesitis, joint deformity, or spinal limitation on physical examination at the time of recruitment. Patients and controls with obesity, diabetes mellitus or metabolic syndrome have been excluded from the study. Clinical assessments, radiographs, and blood samples collection were completed at the study visit. Demographic data, recording of relevant medical history and medications of all the participants were collected. The arthritis activities of PsA patients were assessed by the Psoriatic Arthritis Joint Activity Index (PsAJAI) [11], [12]. The PsAJAI score was calculated as a weighted sum, measuring changes from baselines in the following variables: Joint tenderness count (JTC), C-reactive protein (CRP), Physician global assessment of disese activity (PhGA), Patient global assessment of disease activity (PaGA), patient assessment of pain (PAIN), and Health assessment questionnaire (HAQ). Joint plain radiographs, serum levels of circulating osteoclastogenesis related cytokines and adipokines were also investigated in patients with PsA. And peripheral blood osteoclast precursors were further assessed.

### Radiographic assessments

Plain radiographs of the hands, feet, spine, hip and sacroiliac joints were obtained at the study visit. Plain radiographs of the hands and feet were scored for erosions and joint-space narrowing according to the Sharp van der Heijde score modified for use in PsA by a rheumatologist with experience in this scoring system [13]. The involvements of sacroiliac joints, spine and hip joints were scored as present or absent by a radiologist, according to the Bath Ankylosing spondylitis radiology index (BASRI) [14], [15]. All radiographic scoring and measurement were completed by researchers who were blinded to the clinical and laboratory findings.

### Testing of soluble mediators of circulating osteoclastogenesis related cytokines and adipokines

Blood samples were obtained at the study visit, and serum was separated within 3 hours and stored at −20°C. Expression of the soluble mediators of circulating osteoclastogenesis related cytokines (TNF-α, OPG and RANKL), adipokines (leptin, adiponectin, resistin, chemerin, omentin) in the serum were analysed with enzyme-linked immunosorbent assay (ELISA) by the following kits:

#### RANKL

RANKL concentration was measured by USCN® Human RANKL ELISA Kit , Life Science Inc.USA. The minimum detectable dose of human RANKL is typically less than 0.057 ng/mL. This assay has high sensitivity and excellent specificity for detection of human RANKL. No significant cross-reactivity or interference between human RANKL and analogues was observed. 100 ul serum was used for each test and recovery range was 81–112%, 104% for average. Inter-and intraassay variations were less than 12% and 10%.

#### OPG

OPG concentration was measured by ELISA using USCN® Human OPG ELISA Kit Protocol, Life Science Inc.USA. The minimum detectable dose of human OPG is typically less than 0.061 ng/mL. This assay has high sensitivity and excellent specificity for detection of human OPG. No significant cross-reactivity or interference between human OPG and analogues was observed. 100 ul serum was used for each test and recovery range was 80–116%, 101% for average. Inter-and intraassay variations were less than 12% and 10%.

#### TNF-α

TNF-α concentration was measured by ELISA using USCN® Human TNF-α ELISA Kit Protocol, Life Science Inc.USA. The minimum detectable dose of human TNF-α is typically less than 5.9 pg/mL. This assay has high sensitivity and excellent specificity for detection of human TNF-α. No significant cross-reactivity or interference between human TNF-α and analogues was observed. 100 ul serum was used for each test and recovery range was 99–108%, 105% for average. Inter-and intraassay variations were less than 12% and 10%.

#### Resistin

Resistin concentration was measured by ELISA using XiTang® Human Resistin ELISA Kit Protocol, Shanghai China. The minimum detectable dose of human Resistin is typically less than 15 pg/mL. This assay has high sensitivity and excellent specificity for detection of human Resistin. No significant cross-reactivity or interference between human Resistin and analogues was observed. Diluted serum 1∶20 and use 100 ul of the final diluted serum for ELISA. The recovery range was 95–97%, 96.6% for average. Inter-and intraassay variations were less than 10%. The capture and detection anti-resistin antibody code is ab14051.

#### Chemerin

Chemerin concentration was measured by ELISA using XiTang® Human Chemerin ELISA Kit Protocol, Shanghai China. The minimum detectable dose of human Chemerin is typically less than 2 ng/mL. This assay has high sensitivity and excellent specificity for detection of human Chemerin. No significant cross-reactivity or interference between human Chemerin and analogues was observed. Use 100 ul serum for ELISA. The recovery range was 88–95%, 92.5% for average. Inter-and intraassay variations were less than 10%. The capture and detection anti-chemerin antibody code is ab103153.

#### Leptin

Leptin concentration was measured by ELISA using XiTang® Human Leptin ELISA Kit Protocol, Shanghai China. The minimum detectable dose of human Leptin is typically less than 1.5 ng/mL. This assay has high sensitivity and excellent specificity for detection of human Leptin. No significant cross-reactivity or interference between human Leptin and analogues was observed. Diluted serum 1∶5 and use 100 ul of the final diluted serum for ELISA. The recovery range was 86–100%, 91.8% for average. Inter-and intraassay variations were less than 10%. The capture and detection anti-leptin antibody clone number is 4F8.

#### Adiponectin

Adiponectin concentration was measured by ELISA using XiTang® Human Adiponectin ELISA Kit Protocol, Shanghai China. The minimum detectable dose of human Adiponectin is typically less than 60 pg/mL. This assay has high sensitivity and excellent specificity for detection of human Adiponectin. No significant cross-reactivity or interference between human Adiponectin and analogues was observed. Diluted serum 1∶5000 and use 100 ul of the final diluted serum for ELISA. The recovery range was 97–102%, 100.2% for average. Inter and intraassay variations were less than 10%. The capture and detection anti-adiponectin antibody clone number is 19F1. 19F1 can detect total level of adiponectin including globular adiponectin, low molecular weight, middle molecular weight and high molecular weight adiponectin.

#### Omentin

Omentin concentration was measured by ELISA using XiTang® Human Omentin ELISA Kit Protocol, Shanghai China. The minimum detectable dose of human Omentin is typically less than 1.4 ng/mL. This assay has high sensitivity and excellent specificity for detection of human Resistin. No significant cross-reactivity or interference between human Resistin and analogues was observed. Diluted serum 1∶20 and use 100 ul of the final diluted serum for ELISA. The recovery range was 96–104%, 99.8% for average. Inter-and intraassay variations were less than 9.7%. The capture and detection anti-omentin antibody code is ab101101.

### Cell culture and osteoclast precursors (OCs) identification

Peripheral blood mononuclear cells (PBMCs) were isolated by gradient centrifugation with Lymphoprep (Biowest). The cells were cultured in RANKL and M-CSF as previously described and [16]. PBMCs (10^6^ cells/ml) were seeded in 24-well plates with carry sheet glass containing 1 ml aMEM with 15% fetal bovine serum (FBS), 100 units/ml penicillin, and 100 µg/ml streptomycin. Cells were incubated at 37°C in 5% CO_2_ for 14 days with and without human recombinant RANKL (40 ng/ml; Peprotech Ltd, USA) and M-CSF (25 ng/ml, Peprotech Ltd, USA). Medium was discharged every 3 to 4 days. After culturing for 14 days, slides were stained with TRAP (Sigma, Poole, UK), a widely used marker of osteoclast.

Osteoclasts are characterized by high expression of tartrate resistant acid phosphatase (TRAP) and cathepsin K. TRAP staining, however, remains the most widely used method to describe the formation of osteoclasts in these cultures. Osteoclasts cultured in vitro often being defined as TRAP positive cells with three or more nuclei. Ultimately, phenotypic markers can still only be regarded as suggestive of osteoclastic differentiation as the only definitive marker for osteoclasts remains the ability of these cells to form resorption lacunae (often referred to as pits) on calcified substrates. In this study, we only did TRAP staining which was an imperfect, but well used technique to assess to osteoclast numbers in vitro. However, the gold-standard resorption lacunae will be used in our following studies.

TRAP-positive cells with three or more nuclei were counted as osteoclasts by a single observer who was blinded to the clinical and radiographic characteristics of the patients. For each slide, cells in five sight views which were randomly picked under magnification of 100 times were counted for triplicates. The mean values of each slide counting were calculated as the numbers of OCs.

### Statistical analysis

All data were analyzed by SPSS12.0. Descriptive data are presented as n (percentage) or median (range). Differences between groups was analyzed with χ^2^ tests and Mann-Whitney tests in the case of two groups, and one-way analysis of variance (ANOVA) (Kruskal-Wallis test) with Dunnett multiple comparison test in the case of more than two groups. Spearman's correlations were used to explore the relation between the clinical/radiographic features and laboratory results and followed by linear regressions. A P value of <0.05 was considered significant.

## Results

### Clinical characteristics

Among the 41 patients with PsA, 24 patients had at least one erosions (erosive), 17 patients had no erosions (nonerosive) detected by plain radiography. Clinical characteristics of the healthy controls and the patients with PsA or psoriasis are shown in Table 1. PsA group had longer disease duration and more use of methotrexate and nonsteroidal anti-inflammatory drugs (NSAIDs) compared with the Psoriasis control participants. No patients were receiving TNF inhibitors or other biologic therapy. The level of C-reactive protein and ESR were higher in PsA patients than those in psoriasis patients and healthy controls, but no difference was seen between patients with erosive and nonerosive PsA. Higher PsAJAI, Sharp scores and BASRI were seen in the erosive group compared to the nonerosive group in PsA patients.

**Table 1:** Clinical characteristic of the study participants.

	PsA n = 41	Erosive PsA n = 24	Nonerosive PsA n = 17	Psoriasis alone n = 20	Healthy control n = 24
Female sex, n (%)	15 (37%)	8(33%)	7 (41%)	7 (35%)	8(33%)
Age, years, median (range)	44 (21–62)	41 (35–44)	40 (21–62)	54 (34–65)	43 (33–54)
BMI, median (range)	23 (21–25)	22 (21–24)	23 (22–25)	24 (22–25)	25 (23–27)
Psoriasis disease duration*	148.6±47.3	162.7±25.6	134.5±33.1	56.3±19.6^b^	NA
Arthritis disease duration*	41.5±63.0	54.5±13.0	12.5±4.2^a^	NA	NA
Fasting glucose level*, mmol/L	4.3±0.2	4.2±0.1	4.4±0.1	4.2±0.1	4.3±0.2
C-reactive protein*, mg/L	33.5±34.5	35.4±7.0	29.4±9.0	6.1±3.8^b^	5.2±3.3^b^
ESR*, mm/h	49.8±34.8	49.6±6.2	50.0±11.7	15.3±10.5^b^	12.0±7.2^b^
PsAJAI*	26.6±24.2	32.2±27.7	10.53±8.3^a^	NA	NA
Sharp score*	52.7±40.6	71.7±30.9	3.3±1.3^a^	NA	NA
BASRI*	5.05±2.2	6.5±1.5	3.2±0.8^a^	NA	NA
Methotrexate use, n (%)	22(54%)	15 (65%)	7(41%)	0(0)^b^	NA
Systemic corticosteroids use, n (%)	0(0)	0(0)	0(0)	0(0)^b^	NA
Nonsteroidal antiinflammatory drug use, n (%)	33 (80%)	20 (82%)	13 (76%)	0(0)^b^	NA
Biologics use, n (%)	0 (0)	0 (0)	0 (0)	0 (0)	0 (0)

* Data presented as X±SD;

a P<0.05, compared with erosive PsA;

b P<0.05, compared with all PsA. NA, not assessed.

### Soluble mediators of circulating osteoclastogenesis related cytokines and osteoclast precursors (OCs) in the patients with PsA

**Figure 1:**
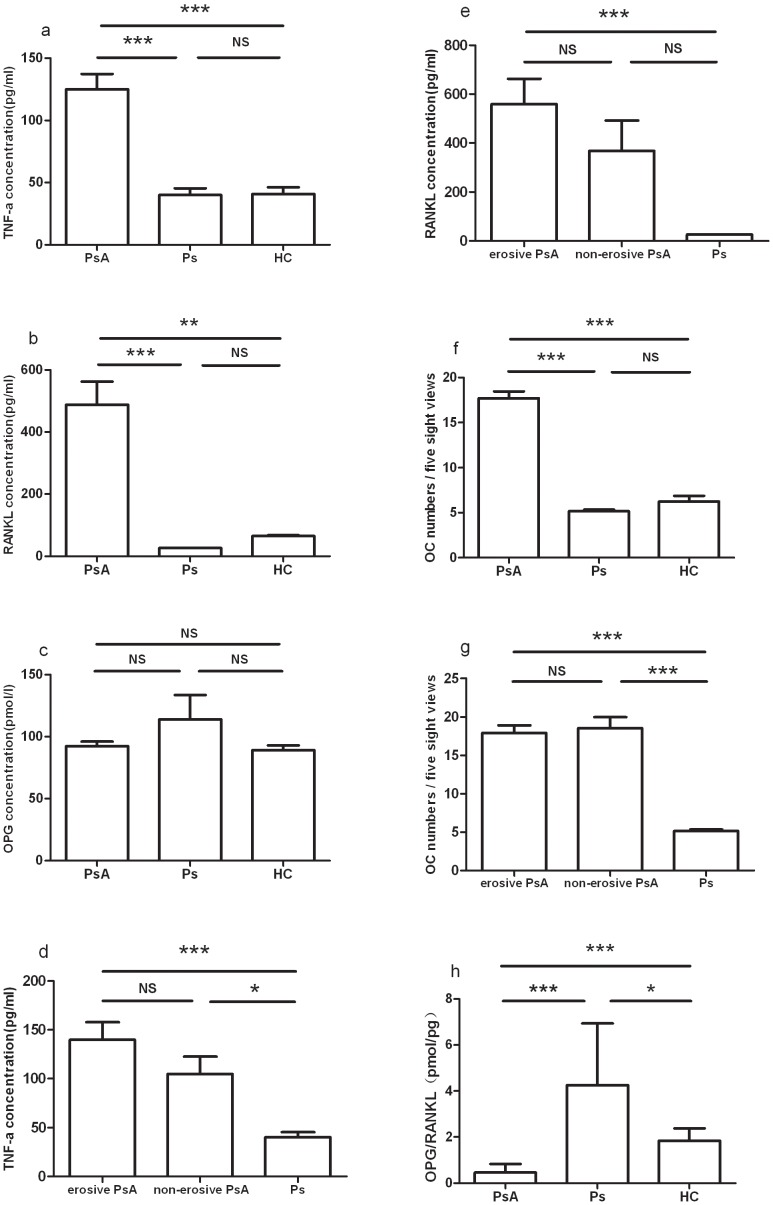
Soluble mediators of bone remodeling in the circulation of patients with PsA. Bar plots show mean concentrations with SEM (standard error of the mean) of (a) TNF-α, (b) RANKL, (c) OPG, (f) OCs and (h) OPG/RANKL (pmol/l to pg/ml) in healthy controls (HCs), patients with psoriasis (Ps) and patients with PsA. Bar plots show mean concentrations with SEM of (d) TNF-α, (e) RANKL, and (g) OCs in patients with psoriasis (Ps) and patients with nonerosive PsA, and patients with erosive PsA. *P<0.05; **P<0.01; ***P<0.001; one-way ANOVA with Dunn's multiple comparison test.

Compared with those in both healthy controls and psoriasis controls, the circulating concentrations of TNF-α, RANKL and OCs in patients with PsA were higher (Figure 1A, 1B and 1F), but the ratio of OPG/RANKL were significantly lower (Figure 1H). No significant difference of OPG concentrations was detected between the control groups and the PsA group (Figure 1C). The circulating concentrations of TNF-α, RANKL and OCs in patients with both erosive and nonerosive PsA were higher than those in the psoriasis controls. No overall difference of TNF-α, RANKL or OCs was noted between the groups of patients with erosive and non-erosive PsA (Figure 1D, 1E and 1G).

### Soluble circulating adipokines in the patients with PsA

**Figure 2:**
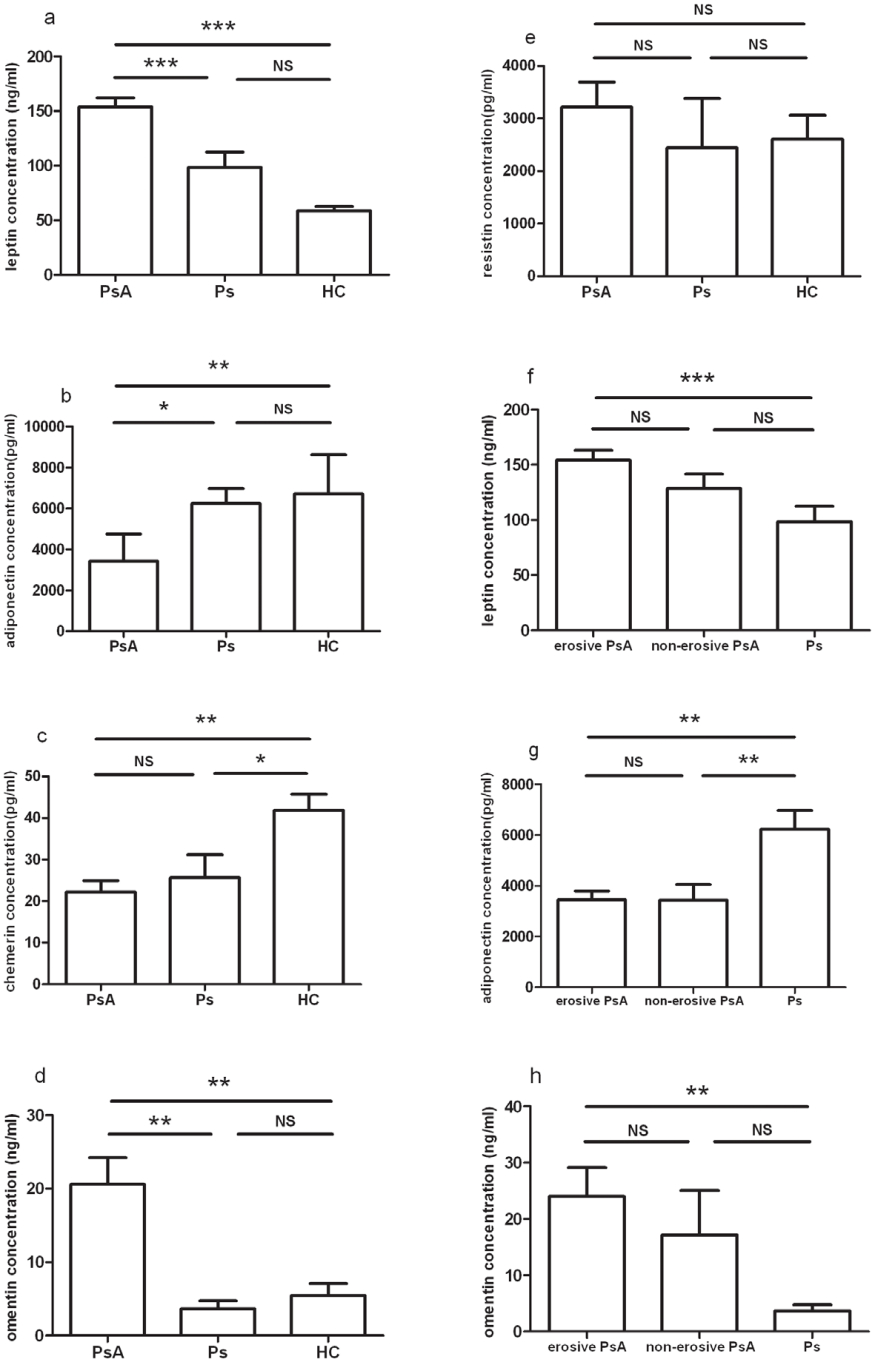
Adipokines in the circulation of patients with PsA. Bar plots show mean concentrations with SEM of (a) leptin, (b) adiponectin, (c) chemerin (d) omentin and (e) resistin in healthy controls (HCs), patients with psoriasis (Ps) and patients with PsA. Bar plots show mean concentrations with SEM of (f) leptin, (g) adiponetin, and (h) omentin in patients with psoriasis (Ps) and patients with nonerosive PsA, and patients with erosive PsA. *P<0.05; **P<0.01; ***P<0.001; one-way ANOVA with Dunn's multiple comparison test.

Compared with those in both healthy and psoriasis controls, the circulating concentrations of leptin and omentin in patients with PsA were higher (Figure 2A and 2D), but the levels of adiponectin were lower (Figure 2B). In addition, chemerin concentration in both Ps controls and patients with PsA were lower than those in healthy controls (Figure 2C). No significant difference of resistin concentration was found between the control groups and the PsA group (Figure 2E). The levels of adiponectin in patients with both erosive and non-erosive PsA were lower than those in psoriasis controls (Figure 2G). In contrast, the concentrations of leptin and omentin in the erosive PsA patients, not in the non-erosive PsA patients, were higher than those in psoriasis controls (Figure 2F and 2H). No overall difference of the concentrations of leptin, adiponectin and omentin was noted between the groups of patients with erosive and non-erosive PsA (Figure 2F, 2G and 2H).

### Peripheral blood osteoclast precursors, soluble mediators of circulating osteoclastogenesis related cytokine, and Adpokines in patients with PsA

In patients with PsA, the circulating concentrations of TNF-α showed positive correlations not only with the number of TRAP+ osteoclast precursors (OCs) after culture with RANKL and M-CSF (r = 0.66; P = 0.000), but also with the radiographic damage scores (Sharp score r = 0.52; P = 0.001; BASRI r = 0.35; P = 0.02 ) and arthritis activity index PsAJAI (r = 0.41; P = 0.02). Both RANKL and leptin were positively correlated with OCs (RANKL r = 0.71; P = 0.000; leptin r = 0.42; P = 0.000) and PsAJAI (RANKL r = 0.44; P = 0.02; leptin r = 0.42; P = 0.03), but not with the radiographic damage scores. Omentin was found to be positively correlated with OCs (r = 0.78; P = 0.000) whereas adiponetin was negtively correlated with OCs (r = −0.57; P = 0.000). Chemerin was only observed to be correlatied with Sharp score, but not with BASRI, OCs and PsAJAI. (see Table 2)

**Table 2:** Correlations among osteoclast precursors, osteoclastogenesis related cytokine, adipokines, arthritis activities and radiographic damage scores in patients with PsA.

	Median(range)	OCs	Sharp Score	BASRI	PsAJAI
TNF-α	125.1(28.2–423.4) pg/ml	0.66^b^	0.52^b^	0.35^a^	0.41^a^
RANKL	488(85.9–1828.6) pg/ml	0.71^b^	0.13	0.28	0.44^a^
Leptin	153.7(81.3–341.1) ng/ml	0.42^b^	0.21	0.15	0.42^a^
Adiponectin	3424.6(693.7–5549.3) pg/ml	−0.57^b^	−0.38	0.05	−0.02
Chemerin	22.2(2.26–83.7) pg/ml	−0.17	−0.35^a^	0.12	0.17
Omentin	20.6(2.8–82.2)ng/ml	0.78^b^	0.34	0.18	0.29
OCs	17.7(7.2–28.5)/5 sight views	——	0.22	0.08	−0.01

a P<0.05;

b P<0.01.

Spearman r values for correlations among Peripheral blood osteoclast precursors, soluble mediators of circulating osteoclastogenesis related cytokine, Adipokines, PsAJAI and radiographic damage scores.

## Discussion

Our study analyzed the correlations among peripheral blood osteoclast precursors, soluble mediators of circulating osteoclastogenesis related cytokine, adipokines, PsAJAI and radiographic damage scores in PsA. The soluble factors analyzed in this study are key regulators of inflammation, osteoclastogenesis and metabolic syndrome, and our data suggested that systemic expression of factors promoting inflammation, metabolic disorders and osteoclastogenesis (TNF-α, leptin, adiponectin, chemerin, omentin, RANKL) were disordered in patients with PsA.

The majority of patients with PsA experience a chronic, progressive destructive, disabling form of arthritis over time. Bone remodeling and osteoclastogenesis are active in these patients. OPG, RANK and RANK ligands are critical molecular determinants of osteoclastogenesis and regulators of bone resorption. In fact, RANKL, a membrane-residing protein on osteoblasts, interacts with RANK, a type I transmembrane receptor present on marrow macrophages, inducing marrow macrophages differentiation into osteoclasts. The main negative regulator of RANKL activity is OPG, which is a soluble decoy receptor for RANKL produced by osteoblasts. This decoy receptor competitively inhibits the binding of RANKL to RANK on the cell membrane of osteoclasts, thus preventing RANK activation and the consequent osteoclastogenesis. The ratio of OPG/RANKL has been widely used to evaluate the bone remodeling and osteoclastogenesis [17]. Our data showed the circulating concentration of RANKL and OCs were significantly higher in PsA compared with those in Ps and healthy controls. The OPG/RANKL ratio was significantly lower in PsA group. RANKL was positively correlated with OCs and PsAJAI. The results provided further evidence for bone remodeling and active RANKL/osteoclastogenesis in patients with PsA. But interestingly, we also investigated that OPG/RANKL ratio was significantly higher in Ps group compared to that in healthy controls. Are there any potential undentified factors protecting Ps patients from osteoclastogenesis? More work need to be done to verify and explain this phenomemon.

**Figure 3:**
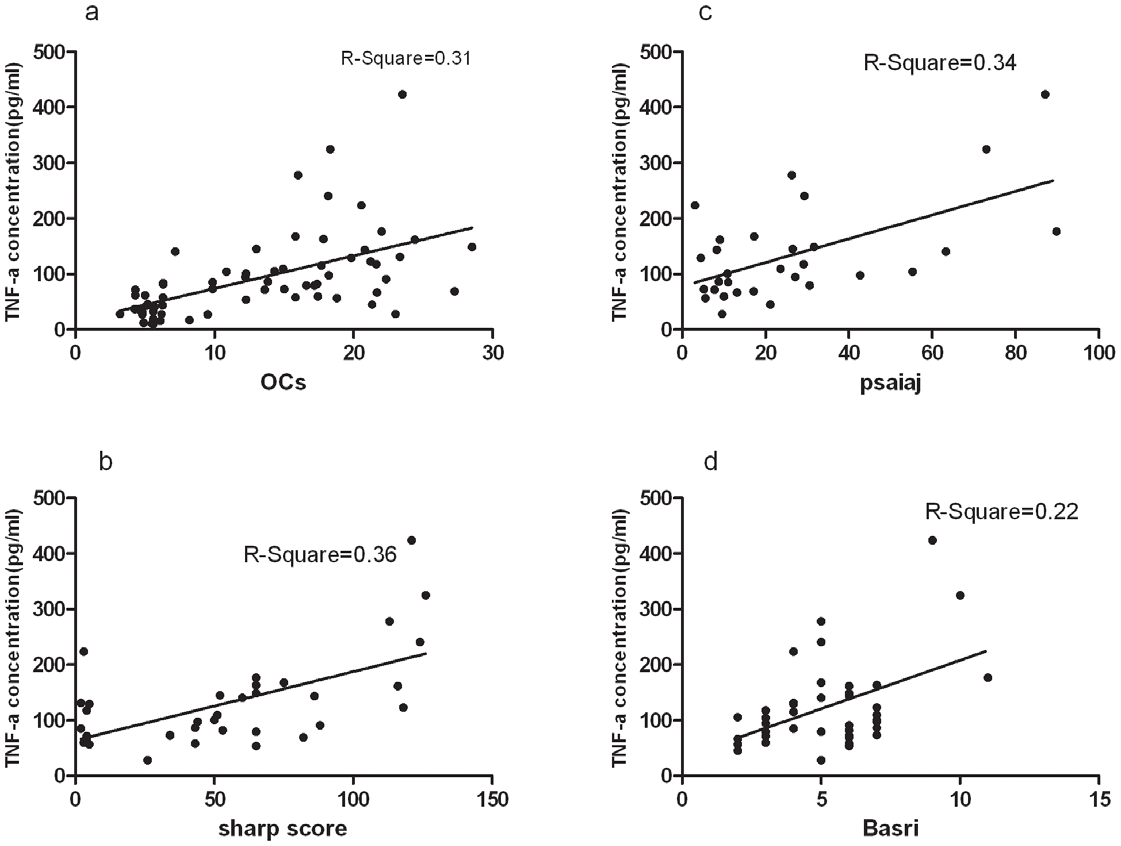
Correlations among serum levels of TNF-α, OCs, radiographic damage scores and arthritis activity index in PsA patients. (a) Serum TNF-α levels positively correlate with OCs. (b) Serum TNF-α levels positively correlate with Sharp scores. (c) Serum TNF-α levels positively correlate with PSAIAJ. (d) Serum TNF-α levels positively correlate with BASRI.

Elevated concentrations of the proinflammatory cytokine, tumor necrosis factor α (TNF-α), have been detected in the joint synovium and lesional skin of patients with PsA [18]–[20].Subsequently, TNF-α has been validated as a therapeutic target in PsA and several other immune-mediated inflammatory diseases. Anti-TNF-α biologic therapies have been demonstrated to significantly reduce the signs and symptoms of PsA [21]–[24]. Our data showed that the circulating concentrations of TNF-α in patients with PsA were higher than those in both healthy controls and psoriasis controls. TNF-α was not only positively correlated with the number of TRAP+ osteoclast precursors (OCs) (r = 0.66; P = 0.000), but also with the radiographic damage scores (Sharp score r = 0.52; P = 0.001; BASRI r = 0.35; P = 0.02) and arthritis activity index PsAJAI (r = 0.41; P = 0.02) (Figure 3A–3D). Our results further supported that TNF-α was a potent cytokine in promoting inflammation and bone erosion. Furthermore, IL-6, IL-17A and IL-23 are also involved in the pathogenesis of PsA and other inflammatory diseases. They are currently being evaluated as therapeutic targets in the treatment of PsA. We tested serum levels of IL-17A, IL-6 and IL-23 in PsA/Ps/healthy controls (data are not shown here) and got significantly higher levels of IL-6 and IL-23 in PsA compared to Ps and HCs. But we failed to detect IL-17A in three groups. More studies focus on these cytokines are needed to be done.

Adipose tissue has long been viewed as a harmless tissue in the pathogenesis of chronic inflammatory connective tissue and joint diseases, with fat providing the soft surroundings for damage inflicted by other mechanisms. However, recent discoveries have completely changed this point of view. First, adipokines are not only produced by adipocytes, but also be produced by various cells of a similar mesenchymal origin such as fibroblasts. Second, the majority of these pluripotent adipokines targets well-known effector cells operative in the pathophysiology of chronic rheumatic diseases towards a proinflammatory and matrix-degrading direction [25].

**Figure 4:**
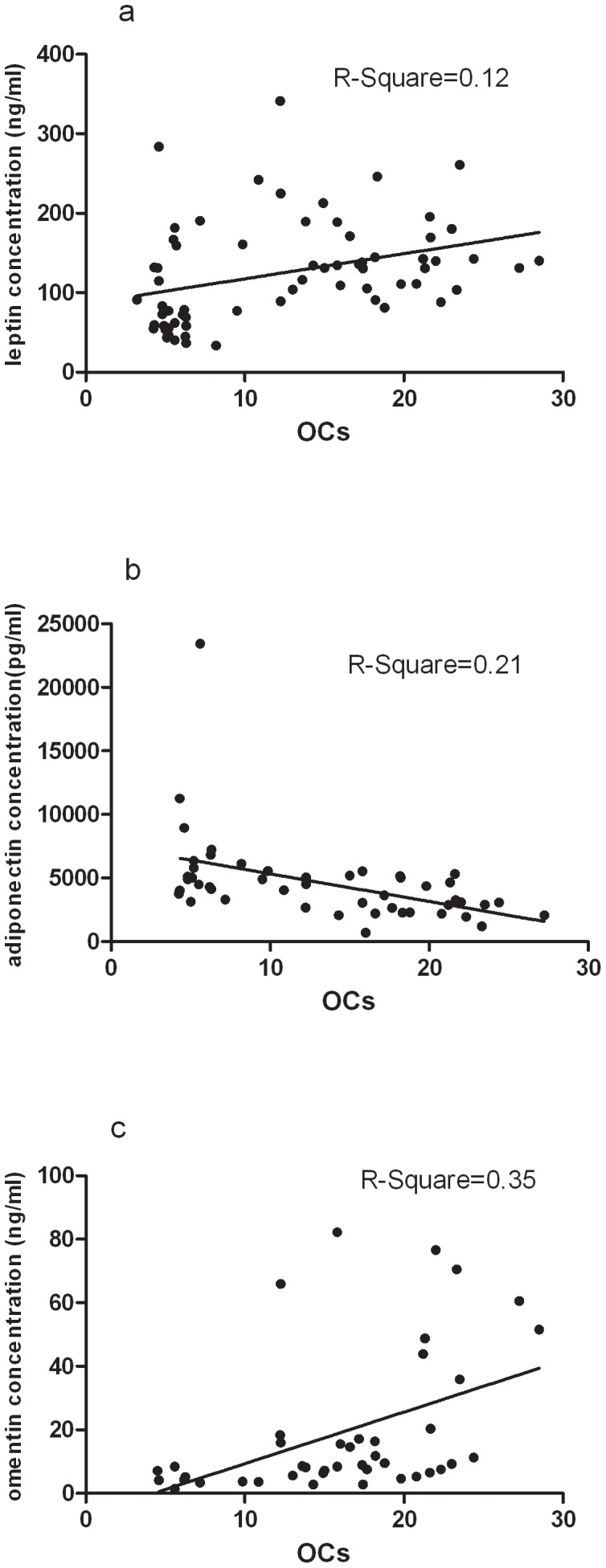
Correlations between adipokines and OCs in PsA patients. (a) Serum leptin levels positively correlate with OCs. (b) Serum adiponectin levels negatively correlate with OCs. (c) Serum omentin levels positively correlate with OCs.

**Figure 5:**
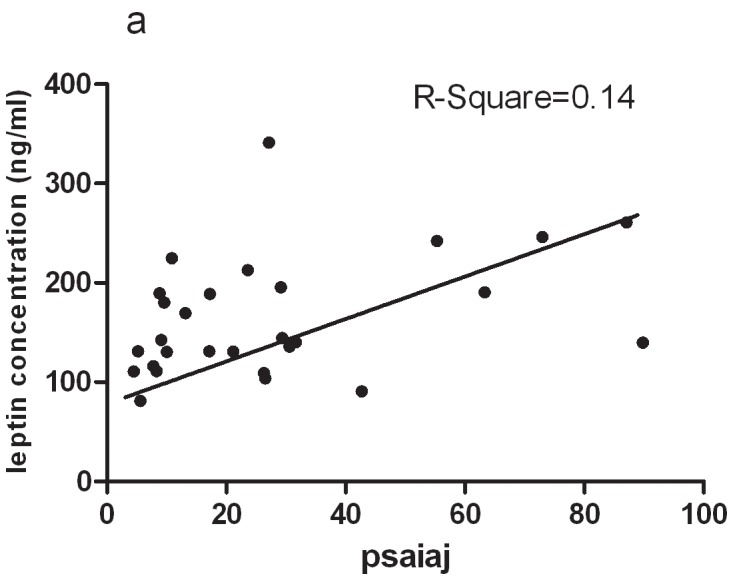
Correlation between leptin and PSAIAJ in PsA patients. (a) Serum leptin levels positively correlate with PSAIAJ.

The adipokine history started with leptin. Leptin has important immunoregulatory functions since it is involved in T-cell proliferation, can induce T-helper type 1 immune reactions, and is involved in proliferation and activation of inflammatory cells such as monocytes and neutrophils [26]. In Cerman's study a significant increase in serum leptin in severely affected psoriasis patients was shown in comparison to mild to moderately affected patients and controls. Assessed by immunohistochemistry, the expressions of leptin and its receptor in skin biopsy samples were only increased in severely affected psoriasis patients. In addition, serum leptin levels, tissue leptin and leptin receptor expression showed a positive correlation with disease duration in patients with psoriasis The authors concluded that leptin might serve as a marker of severity and chronicity in psoriasis [27]. In Otero's study, a marked increase levels of leptin in plasma was noted in patients with rheumatoid arthritis [28]. Leptin was observed in Synovial fluid obtained from human OA-affected joints, and the leptin concentrations was positively correlated with the body mass index. Marked expression of leptin was observed in OA cartilage and in osteophytes, while in normal cartilage, leptin was only detected in few chondrocytes. Furthermore, the pattern and level of leptin expression were related to the grade of cartilage destruction and paralleled those of growth factors (Insulin-like Growth Factors-1 and Transforming Growth Factor β-1). These findings suggested a new peripheral function of leptin as a key regulator of chondrocyte metabolism, and indicate that leptin may play an important role in the pathophysiology of OA [29]. Leptin also plays a role in bone metabolism. Leptin regulates energy metabolism, reproduction and bone mass accrual through inhibiting serotonin (5-hydroxytryptamine) synthesis and release by brainstem neurons. In our study, the increased levels of leptin in PsA patients were 153.7(81.3–341.1)ng/ml, which were much higher than those in Chinese obesity population (16.59±6.92)ng/ml. In addition, leptin was also positively correlated with OCs and PsA disease activity index (Figures 4A and 5A). These findings suggested that leptin played a role in the exacerbation of PsA. Chronic inflammation status may contribute to higher levels of leptin which in turn exacerbates the inflammation leading to bone remodeling. Functional or mechanistic data is necessary to support such hypothesis.

Adiponectin is a next widely studied adipokine. It exists in various isoforms with different and sometimes counteracting functions. In a study of adiponectin in psoriasis patients, serum adiponectin levels were positively related to Psoriasis area and severity index score (PASI). In contrast, serum high molecular weight (HMW) adiponectin levels were decreased in psoriasis patients and negatively correlated with PASI [30]. Moreover, globular adiponectin strongly inhibits TNF/RANKL-induced osteoclastogenesis as well as osteoclast formation induced by innate immune system arthritis-related Toll-like receptor 4 ligand and RANKL. It is possible that the biological effect of adiponectin could be dissected based on its molecular weight and formation. In our study, toltal level of adiponectin included globular adiponectin and HMW adiponectin. The results showed that the level of toltal adiponectin was significantly lower in patients with PsA than those in both healthy and psoriasis controls. In addition, adiponetin was analysed to be negatively correlated with OCs (Figure 4C). These findings of adiponectin supported the that globular adiponectin strongly inhibited TNF/RANKL-induced osteoclastogenesis as well as osteclast formation. Further studies should be done to verify the different biological effects of adiponectin based on its molecular weight and formation in the pathogenesis of PsA.

Chemerin, known as tazarotene-induced gene 2 and retinoic acid receptor responder 2 (RARRES2), is a novel identified chemoattractant adipokine [31]. Chemerin acts via the G-coupled receptor chemokine-like receptor 1 (CMKLR1 or ChemR23) [32]. Chemerin and its receptor are mainly located, but not exclusively, in adipose tissue [33]. For instance, dendritic cells and macrophages express chemerin receptor [34]. ChemR23, also expressed by endothelial cells, is upregulated by proinflammatory cytokines such as TNF-α, IL-1β, and IL-6 [35]. Interestingly, chemerin and its receptor are also located in chondrocytes [36]–[38]. And IL-1β is able to induce chemerin upregulation [37]. In the same way, It has been demonstrated that recombinant chemerin enhanced the production of several proinflammatory cytokines (TNF-α, IL-1β, IL-6, and IL-8), as well as different MMPs (MMP-1, MMP-2, MMP-3, MMP 8, and MMP-13) in human articular chondrocytes [36]. These factors play a role in the degradation of the extracellular matrix and result in the irreversible destruction of the cartilage in OA and RA by causing a breakdown of the collagen and aggrecan framework. Moreover, It was reported that the intracellular signalling after ChemR23 activation occurs through p42/44 MAPK and Akt phosphorylation. Evidence was provided that chemerin, acting through the CMKLR1 receptor, played a critical role in promoting the adipogenic differentiation of bone precursor cells and negatively regulating osteoblast differentiation [39]. In our study, chemerin was detected to be significantly lower in the Ps and PsA patients than that in the healthy controls. The expression of chemerin in inflammation areas (eg. synovial fluid/tissue of PsA, skin lesions of Ps) are necessary to be further studied.

Omentin is a protein of 40 kDa secreted by omental adipose tissue and highly abundant in human plasma. It was previously identified as intelectin. It was suggested that a biological function of omentin/intelectin was the specific recognition of pathogens and bacterial components, playing an important role in the innate immune response to parasite infection [40]. Moreover, studies have shown that omentin gene expression is altered by inflammatory states and obesity [41]. Intriguingly, a differential expression of omentin mRNA occurs in omental adipose tissue of patients with Crohn's disease, suggesting that omentin could be a new candidate factor potentially involved in chronic inflammatory diseases in humans [42]. Recently, Senolt et al. found reduced levels of omentin in the synovial fluid of patients with RA compared to those with OA [43]. This finding suggests that omentin is likely involved in OA pathophysiology. In our study, omentin was significantly increased in patients with PsA compared with both healthy and psoriasis controls. This result differs from the work mentioned above might because of the different sources of samples in our study. Replicated work should be done to demonstrate that whether the omentin concentration in serum is differ from the synovial fluid. In addition, we found omentin was positively correlated with OCs (Figure 4B).

Resistin,another powerful adipokine,is synthesized in the lining layer by macrophages, B cells, and plasma cells. All these cells are strongly operative in rheumatoid pathophysiology [44]. Corbetta and co-workers showed that increased serum resistin levels in untreated psoriasis patients were normalized after 1 and 3 months of acitretin therapy [45]. Serum resistin levels have been shown to be higher in patients with RA than those in healthy controls, although no differences between sexes have been reported. Resistin levels also correlate with inflammation, joint destruction and levels of IL-1 receptor antagonist in women with RA [46]. In RA, macrophages, B lymphocytes and plasma cells, but not T lymphocytes, showed co-localization with resistin [47]. Resistin levels in synovial fluid and serum were higher in RA patients than those in OA patients, and positively correlated with both C-reactive protein (CRP) levels and 28-joint disease activity score (DAS28), but not with levels of other adipokines. Notably, RA patients treated with infliximab showed a rapid reduction of serum resistin levels which is in close correlation with levels of CRP and other markers of inflammation [48]. The role of resistin in cartilage has also been studied, particularly in the patients with joint lesions. Resistin is elevated both systemically and locally in weeks immediately after joint injury, and has a direct effect on cartilage matrix turnover and cytokine production. However, resistin levels gradually declined post injury over time [49]. Sandell et al. [50] demonstrated that resistin had diverse effects on the expression of chemokines, cytokines, and matrix genes in human chondrocytes via mRNA stabilization and transcriptional upregulation. In our study, no significant difference of resistin concentration was detected between the control groups and the PsA group. Replicated work need be done to verify the role of resistin in PsA.

In this study, joints destruction was assessed with plain radiography using a widely recognized scoring method of established bone change. Although no correlation between circulating bone remodeling markers or adipokines and Sharp score or BASRI was observed, it is possible that inflammation of the joints was underestimated using this method, compared with a more-sensitive method such as magnetic resonance imaging. Chemerin was the only adipokine observed to be negtively correlatied with Sharp score, but not with BASRI, OCs and PsAJAI. Further investigations are needed to explain these conflicting results.

The key finding of this study is the elevated serum leptin concentrations in PsA patients which were correlated positively with OCs and PsAJAI (Figures 4A and 5A). PsAJAI is a new scoring tool designed to assess the response rate of patients with active PsA. These findings strongly implied that leptin might implicate in joint remodeling in inflammatory arthritis, blockade of this factor might inhibit osteoclastogenesis and bone erosion in joint inflammation. Leptin might serve as a marker of severity in psoriatic arthritis patients. Adipocytes in the environment of local joint, perhaps altering osteoblast function or expressing of proinflammatory cytokines or adipokines, may act in concert with soluble mediators of bone remodeling such as RANKL to promote osteoclastogenesis, and in turn bone erosion. Our data support a potential role of leptin, adiponectin and omentin in modulating osteoclast precursors. Further basic researches should be done in the regulatory function of adipokines on osteoclastogenesis and osteoclasts.

## Conclusions

This study suggested that systemic expression of soluble mediators of osteoclastogenesis and adipokines were disordered in PsA. Certain adipokines were elevated in the circulation of patients with PsA and might contribute to pathogenesis of arthritis. Prospective studies will be of interest to determine the pluripotent effects of adipokines on chronic inflammatory rheumatic diseases, as well as their role on bone matrix remodeling. Future studies may lead to novel therapeutic strategies.
